# Enhancing Durability and Efficiency of Electrochemical Energy Devices (Batteries and Solid Oxide Cells) Through Thermal Spray Coating Technologies: A Review

**DOI:** 10.3390/ma19153175

**Published:** 2026-07-24

**Authors:** Amrinder Mehta, Hitesh Vasudev, Brijesh Prasad, Suresh Singh, Manoj Kumar, Sachin Sirohi

**Affiliations:** 1 Research and Development Cell, Lovely Professional University, Phagwara 144411, Punjab, India; amrindermehta896@gmail.com; 2 Department of Mechanical Engineering, Graphic Era (Deemed to be University), Dehradun 248005, Uttarakhand, India; prasadbrijesh10@geu.ac.in; 3 Centre for Research Impact and Outcome, Chitkara University Institute of Engineering and Technology, Chitkara University, Punjab, Rajpura 140401, Punjab, India; sureshsinghcu87@gmail.com; 4 Department of Mechanical Engineering, ABES Engineering College, Ghaziabad 201009, Uttar Pradesh, India; mky2011@gmail.com; 5 Department of Mechanical Engineering, SRM Institute of Science and Technology, Delhi NCR Campus, Modinagar 201204, Uttar Pradesh, India

**Keywords:** thermal spray coatings, electrochemical energy devices, batteries, solid oxide cells, fuel cells

## Abstract

**Highlights:**

**Main findings**

- Coatings of thermo spray increase electrochemical stability and battery resilience.
- Transport and degradation are controlled by the microstructure (porosity, splats, and phases).
- HEAs, FGMs, and nanostructures make performance-customized interfaces.
- The balance of energy in the process of powder synthesis is connected with the performance of the coating.

**Implications of the main findings**

- The use of longer-life energy devices is made possible by interface-engineered coatings. Scalable battery manufacture is assisted by design-based spray techniques.
- The optimization and reliability of the coating are enhanced by AI and modeling.
- Favors the next-generation batteries and solid oxide.

**Abstract:**

Thermal spray coatings have also been adopted as a solution strategy to increase the service life and improve the performance of designed engineered electrochemical energy devices, especially batteries and solid oxide cells. These systems share common attributes of challenges in interfacial stability, degradation in microstructure, and transport limitations in extreme operating conditions. This review revolves around the significance of microstructural coating, that is, distribution of porosity, phase composition, splat bonding, and interface quality in regulating the electrochemical performance. High-entropy alloys, functional graded material, and nanostructured feedstock are also mentioned in the context of how they can be utilized to modify coating properties and make the device more reliable. The processing environments and microstructure development relative to major performance indices, such as ionic/ electronic conductivity, corrosion behavior, and stability, are critically examined. Furthermore, the trade-off between the energy consumed during the synthesis of the powders and the efficiency of deposition of the coating is discussed to give information on sustainable production. Other new approaches involving the use of computational models and artificial intelligence to maximize the processes are also discussed in this review. Generally, this paper creates a process–structure–performance system for the rational development of thermal spray coatings in the next generation of electrochemical-based energy systems.

## 1. Introduction

Electrochemical energy devices, such as batteries and solid oxide cells, are critical for addressing global energy challenges and achieving net-zero emissions. These devices, however, face significant challenges related to interfacial instability, degradation, and performance under extreme operating conditions. The major issues are interfacial unsteadiness, overpotentials, charge transfer inefficiencies, capacity fading, and stability issues over the long term due to interactions of electrode and electrolyte microstructures and microstructural evolution. The currently used coating and surface modification technologies frequently cannot provide a trade-off between scalability, durability, and electrochemical performance, particularly in complicated multi-layered structures and novel battery chemistries [1,2,3,4,5]. These difficulties demonstrate the importance of determining electrochemical performance by electrode surfaces and interfaces. This is also true of battery systems, where charge transfer, capacity retention, and long-term stability are determined by electrode–electrolyte interactions. Both instances are also strongly associated with interfacial reactions, microstructural evolution and transport restrictions [6].

Thermal spray coatings can significantly extend electrode lifetime by modifying surface properties and improving interfacial connectivity. With thermal sprays, it is possible to get surface property modification and improved connectivity. Such coatings enhance efficiency and durability by controlling key microstructural features such as porosity and interfacial bonding [7]. This cross-system perspective forms the basis for applying thermal spray coating strategies across different electrochemical technologies.

### Analysis of Technology Methods

Electrode performance and response time in battery systems were assessed using electrochemical impedance spectroscopy (EIS), which contributes to understanding the charge transfer resistance and ion diffusion mechanisms that affect battery performance. Cyclic voltammetry (CV) was used to analyze electrode surface reactions, which are critical to the study of electrochemical activity and stability when used in batteries [8]. In battery system scenarios, the best imaging techniques, such as scanning electron microscopy (SEM) and transmission electron microscopy (TEM), were used to analyze the shape and location of the catalysts on the electrodes, providing comprehensive structural information that determines electrode performance and lifespan. In situ spectroscopic studies, such as Raman spectroscopy and X-ray absorption spectroscopy (XAS), provided real-time thermodynamics of the electrolyte in battery operation. These enhanced characterization techniques have helped to increase the efficiency, stability, and scalability of battery systems through the optimization of catalyst design and enhancement of electrode–electrolyte interactions [9,10].

Thermal spray coating (TSC) technology is a scalable, flexible and design-driven technology to increase the efficiency and durability of electrochemical energy devices. One of the strengths of TSC is the fact that coating microstructure, which includes porosity, phase composition, splat bonding, and interfacial quality, can be precisely tailored, and this directly controls the ionic/electronic conductivity, gas permeability and mechanical integrity. As an example, recent studies by Kiape et al. have revealed that CoCrFeMnNi_0.8_V/Cr_3_C_2_–Ni20Cr high-entropy alloy composite coatings have better hardness (~9–11 GPa) and wear resistance than monolithic coatings, and compositional design and phase distribution have been shown to be important in enhancing coating stability [11]. In this regard, material design of the next generation, including functionally graded materials (FGMs) and high-entropy alloys (HEAs), has become an enabling technology for designing next-generation electrodes and interfaces. Sathish et al. showed that FGMs produced through atmospheric plasma spraying have controlled porosity gradients (≈10–30%), which efficiently minimize thermal and mechanical mismatch at interfaces, subsequently leading to stronger adhesion strength and higher cycling stability with repeated electrochemical loading. Shang et al. also discussed in the framework of electrochemical systems that microstructures and interfaces of electrode coatings are also essential to enhance electrochemical performance, flexibility, and charge transport. TSC can prevent corrosion and wear, extend the life of devices, and ensure the continued functioning of devices under severe operating conditions by improving the properties of electrode surfaces, interfacial bonding, and extending the operating life of the device. Moreover, compared with traditional coating methods, TSC processes have unique features, such as scalability in industrial manufacturing, economic effectiveness, and deposition of thick, nanostructured layers depending on the needs of a particular component of the battery and its functionality [12].

Atmospheric plasma spraying (APS) is the most frequently used thermal spray technique in electrolysis, and it is attributed with flexibility and low cost to form functional electrode layers. Its applicability has been shown in alkaline and PEM systems, especially when it has been found to be excellent in the formation of porous electrode structure [13]. Vacuum Plasma Spraying (VPS) is a technology used to create high-quality coatings. It uses a controlled atmosphere to do this. In particular, it is useful for coating titanium bipolar plates in PEM electrolyzers. Spray coating is done in a vacuum. This stops oxidation, creating a strong coating. The coating protects well against rust and conducts electricity well [14]. This is possible using the well-developed suspension plasma spraying (SPS) processes in which nanostructured coatings are obtained by increasing the surface area of the coating and increasing the catalytic ability. This method can control the structure and makeup of the coatings. This is especially good for the electrochemical activity of alkaline electrolyzer electrodes [15].

Table 1 reveals that TSC has been widely studied in electrochemical devices, including batteries and solid oxide cells, in which other interfacial transport, degradation, and microstructural issues are similar. However, knowledge gained in related electrochemical systems (e.g., electrolyzers and solid oxide cells) is considered only where it offers transferable information about the coating behavior and interface stability of batteries. Other than thermal spray methods, other methods of coating like ALD, CVD, and polymer-based coating come in to be compared to show the differences in scalability, interfaces control, and electrochemical performance.

Even though there are studies that have a basis in electrolysis and solid oxide systems, they have been included because of the existence of common process–structure–performance relationships that are directly relevant to battery interfaces. Increasing the temperature increases the kinetics of the electrode reactions, thus increasing the efficiency of the process owing to reduced activation barriers. However, the ohmic and concentration overpotentials are likely to increase and require careful optimization [33]. Despite these advantages, high-temperature electrolysis faces certain challenges, including material degradation and the requirement for high-value materials that can tolerate extreme conditions. Only the cost effectiveness of high-temperature electrolysis is being determined, with the costs at present not being considered competitive without government subsidies. High-temperature electrolysis uses less energy and reduces electricity needs. But it has problems with material durability and cost. To produce hydrogen on a large scale, we need to find a balance between faster reactions and the extra costs needed to keep things running [34,35,36,37].

**Figure 1:**
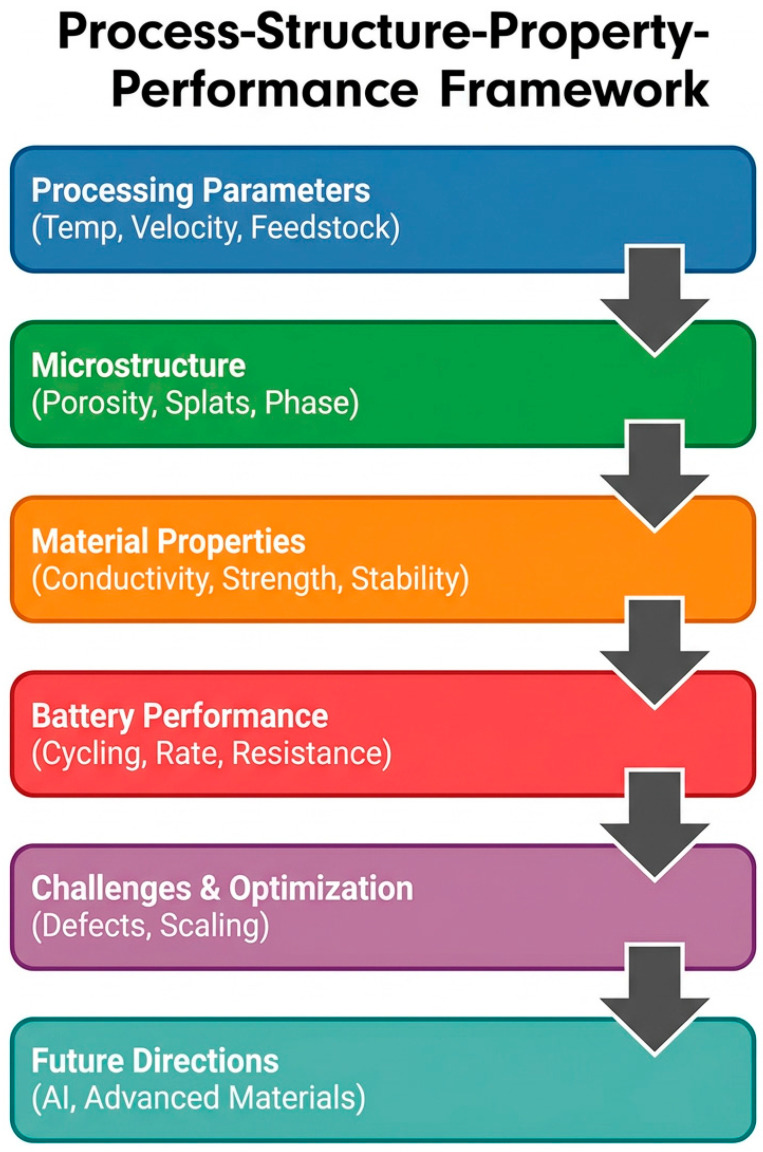
Flow diagram of the process, structure, property, performance, and chain in battery materials.

Figure 1 shows a sequence from left to right. The size and shape of the particle are determined by the method of making the powder. This influences the coating structure, such as splats, holes and surfaces. The microstructure regulates properties such as conductivity, resistance to corrosion, and strength. These properties influence the performance of the battery, such as its cycle life, rate capability, and stability over time. Performance metrics such as cycling stability, rate capability, and interfacial resistance are directly governed by the coating microstructure.

This review reflects on thermal spray coating technologies in three primary electrochemical systems, which include batteries and solid oxide cells. They are concerned with their shared issues at interfaces, wear out, and performance limits. This review brings out the similarities between the process, structure, and performance rather than looking at the individual systems. In contrast to past reviews, this work provides (i) a quantitative comparison of thermal spray processes in the form of structured tables, (ii) a distinct separation of coating characteristics and electrochemical performance indicators, and (iii) an integrated perspective involving experimental, computational, and data-driven research.

## 2. Fundamentals of Thermal Spray Evolution

The goal of the right combination of advanced processes and materials used in the synthesis of thermal spray coatings for electrochemical energy applications is to develop electrochemical devices (batteries and supercapacitors) with enhanced performance. This is done to exploit the nature of TCS [38,39,40]. Electrochemical thermal spraying is a complex field of study. Electrochemical thermal spraying is a complex field that focuses on the type of material used, coating methods, and understanding how coatings work electrochemically. They are used in harsh electrochemical conditions, such as those of increased oxidation resistance and mechanical strength, with such materials. The incorporation of yttrium oxide in thermal spray surfaces has demonstrated remarkable properties regarding plasma etching, which is highly valuable in maintaining the integrity of the coating in electrochemical applications [41,42]. It has been demonstrated that hybrid ceramic and fluoro-silicon sealing materials have superior electrochemical characteristics and therefore enhance the performance and longevity of corrosive environment coating.

**Figure 2:**
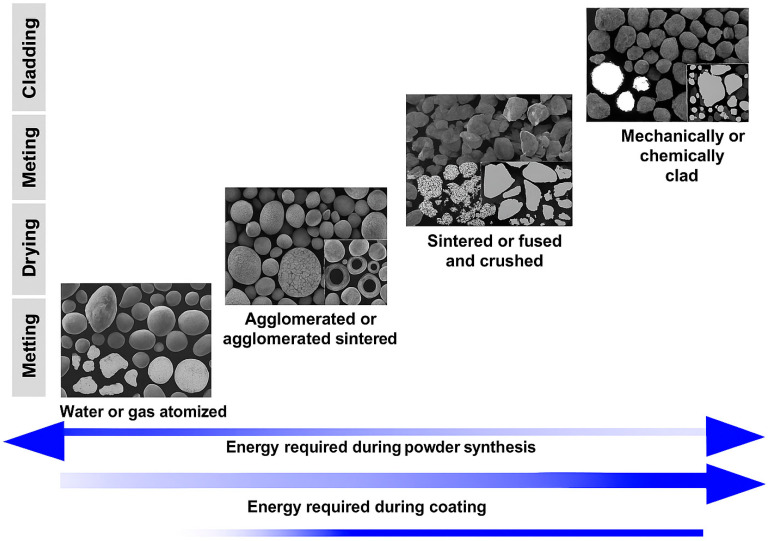
The processes of the synthesis of the powder and thermal spray are mutually dependent with regard to the energy consumption [43].

Figure 2 shows close-up images of the different methods of thermally coating synthesis in terms of the power requirements of each process during the synthesis of powders and the coating process. The diagram identifies four different processing paths: water or gas atomization, agglomeration or agglomerated sintering, sintering or fusion and crushing, and mechanically or chemically clad powder preparation. Scanning electron microscopy (SEM) images characterize each method, showing the typical morphology of the particles produced by the various synthesis methods. The arrows at the bottom show that more energy is needed for higher energy demands when making the powder. Water or gas atomization uses the least energy, while mechanical or chemical cladding uses the most energy. On the contrary, the energy needs of coating are of an inverse pattern, in which water-atomized powders require a lot of energy in the thermal spray process compared to pre-processed powders. The depicted microstructures of the particles show how the various synthesis processes create varying levels of internal porosity, surface roughness, and density of the particles, which directly influence the coating deposition behavior and resultant coating properties. The energy needed to make the powder and the energy needed to apply the coating are connected. If more energy is used to make the powder, less energy is needed to apply the coating. This balance is important in thermal spray processing. There is also a chance that using more energy to make the powder can improve the quality and performance of the coating [43].

The thermal spray coating phase composition and microstructure control the electrochemical properties of the thermal spray coating, which can be optimized by selectively choosing feedstock materials and spray parameters. The energy needed to synthesize the feedstock powder depends on a number of factors, such as the method of synthesis, the size distribution of the particles, as well as the contact between the particles and binders [44,45,46].

**Figure 3:**
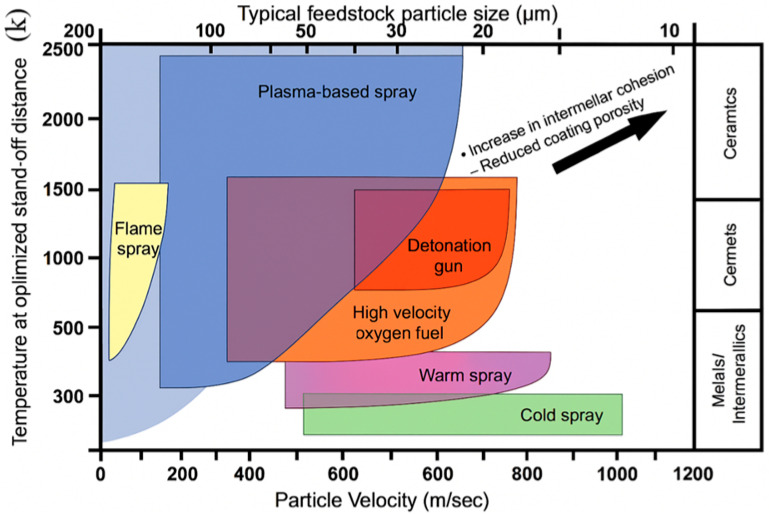
Sorting of the thermal spray processes based on the velocity of the particle, temperature of the particle and the average size of the feedstock [47].

Figure 3 shows four ways to make the powder: water or gas atomization, agglomeration (or agglomeration-sintering), sintering (or fusion and crushing), and mechanical/chemical cladding. These methods are arranged along an energy line. Powder production is on the left, and coating applications are on the right. Water and gas atomization occur in the low-energy range during powder synthesis, which produces dense and spherical particles. When more energy is used for making materials, the particles stick together in uneven shapes with controlled holes. Mechanical or chemical methods need the most energy to create mixed or layered powders. Conversely, powders produced with low energy input (e.g., atomized powders) require a large amount of thermal spray energy to fully melt and deposit. Modern pre-made powders use less energy for spraying. This means that they save energy when making powders but use more energy when applying coatings. These factors affect how the material melts, how well it sticks, and the final coating’s structure [47].

Different synthesis processes and material characteristics can have a great impact on the energy consumption and feedstock production efficiency. Understanding these relationships is necessary to optimize manufacturing procedures and ensure the sustainability of material production. Although the environmental impact of these processes and sustainability should also be considered, the energy consumed during feedstock powder synthesis is a key factor. Recycling and reusing unused powder are some of the methods that could be used to minimize the use of resources and to increase the sustainability of the thermal spray coating operations [48]. The present review focuses mainly on thermal spray coating technologies because they are scalable and universal methods for improving electrochemical energy devices. Additional coating methods, including atomic layer deposition (ALD), chemical vapor deposition (CVD), and polymer-based coatings, are also mentioned to provide a comparative value on the appropriateness of thermal spray methods. The focus of this study is on the impact of thermal spray processing, microstructure, and material selection on the electrochemical performance and durability.

## 3. Applications in Electrochemical Devices

### 3.1. Critical Comparison

Various thermal spray methods have strengths and weaknesses applied to battery systems, based on the desired coating characteristics, microstructure, and operational requirements. Atmospheric plasma spraying (APS) is widely used because of its versatility and ability to deposit ceramic coatings with controlled porosity and phase composition. It provides good control over porosity and phase composition; however, oxidation and poor inter-splat bonding may restrict the electrical conductivity of battery electrodes. APS is more applicable to cathode coatings and protection oxides [49]. HVOF spraying is used to produce dense coatings with high adhesion strength since high velocities of the particles are used. It has better mechanical integrity and reduced porosity than APS; hence, it is applicable to current collectors and transport layers. Nonetheless, it is not very efficient in depositing highly porous (or nanostructured) coatings needed in certain battery electrodes [50]. Cold Spray is relatively low temperature, and thus oxidation and phase decomposition are prevented. It yields very dense and ductile surfaces with good adhesion, which is useful in steel current collectors and metallic conductive layers. The limitation of the method, however, is the deposition of ceramic materials and controlled porosity [51]. Suspension Plasma Spraying (SPS) allows nanostructured and highly porous surfaces to be produced with increased surface area and is therefore especially useful when developing electrochemically active battery electrodes. However, phase stability and control of the process are not easy [52].

A comparison of thermal spray techniques in the use of electrochemical energy is indicated in Table 2. It contains research from significant journals. The results revealed the relationship between the pro-processing procedures and the control of microstructures and performance. Durability and stability can be enhanced by such advanced techniques as HEA-based coating and graded materials. Suspension and solution precursor techniques help control tiny features important for ionic transport.

Extremely important to the structure and performance of the coating is the high-velocity oxygen fuel (HVOF) spraying configuration, such as the range of the spray, the proportion of oxygen and fuel. These settings are the ones that when correct allow the coating to be less porous, denser, stickier, and tougher. This enhances the wear and heat resistance, which is essential in a rugged battery environment [74,75].

### 3.2. Battery Systems

Surface coating of the electrode materials has emerged as a mandatory course to the advancement of battery technologies, particularly lithium-ion, sodium-ion, and all-solid-state batteries. These coatings serve multiple functions, including enhancing electrochemical activity, improving mechanical stability, and mitigating interfacial degradation. They can improve electrochemical activity by increasing the material’s conductivity and strength. They help the material last longer by slowing down wear and tear. They also prevent damage where the electrode and electrolyte meet, which helps keep the capacity stable [76]. Coating cathode materials with thermal spray techniques improves electrochemical activity and mechanical robustness. The metal oxides Al_2_O_3_, TiO_2_, ZrO_2_, HfO_2_, phosphates, borates, and conductive polymers are commonly used coatings that stabilize cathode surfaces, suppress unwanted side reactions, and enhance cycling stability [77].

The purpose of Al_2_O_3_ coatings is to provide a physical barrier that stabilizes the interface of the solid electrolyte (SEI) and reduces capacity fading by preventing direct contact between the electrode and electrolyte. TiO_2_ coatings may be used in a dual mode as protective barriers and electrochemically active materials to enhance thermal safety and energy density. ZrO_2_ coatings are thermally stable and mechanically strong, which promotes ion transport and suppresses electrolyte breakdown. HfO_2_ coatings are highly stable for long cycles because of their chemical stability and enhanced retention of capacity and internal resistance [78,79,80]. These coatings are particularly effective for high-nickel cathodes, where they reduce metal dissolution and mechanical degradation during cycling. The TiO_2_ coating is effective in terms of its capability to act as both a protective barrier material and an electrochemically active material by lithiation. The TiO_2_ coating inhibits exothermic reactions at high rates in lithiated silicon anodes and enhances the thermal safety by far, with only a slight decrease in electrochemical performance. Research has indicated that TiO_2_-silicon anodes have a 30% higher volumetric energy density than pure materials [81]. The fabrication of dense and gas-impermeable layers of electrolytes through thermal spray coating allows the microstructure to be optimized to facilitate ionic conductivity and mechanical integrity. Thermal spray is used to create multilayer ceramic structures, including yttria-stabilized zirconia (YSZ) and lanthanum strontium manganite (LSM) which are used to create dense electrolytes with graded compositions that minimize thermal mismatch and enhance electrochemical performance. The porosity and phase composition of these coating are important to be controlled to achieve gas tightness and also to reduce the degradation of the coating when subjected to operating conditions. Inter-splat bonding and densification are improved by post-spray sintering to enhance the performance of electrolytes [82,83,84].

Interconnects offer the advantage of thermal spray, which offers corrosion-protective qualities, electrical conduction, and mechanical longevity. Plasma spraying titanium substrates has been used to apply coating to the electrolyte surface of proton exchange membrane (PEM) electrolyzers to minimize oxidation and enhance electrical contact. Coating in controlled atmospheres will eliminate oxidation to achieve high splat bonding and low interfaces resistance. High-entropy alloys and functionally graded materials used as interconnect coatings improve stress tolerance and long-term stability by eliminating thermal expansion discontinuities and phase segregation [14,85,86,87].

The electrochemical performance of LiMn_2_O_4_ (LMO) and its aluminum-coated variant (LMO@Al) has been extensively investigated to enhance the stability and capacity of lithium-ion batteries. LMO is an attractive cathode material due to its large specific capacity; LMO has the disadvantage of being structurally unstable in the process of cycling. By coating LMO, unwanted manganese dissolution is eliminated, and the charge-transfer resistance decreases, thereby improving the electrochemical performance of LMO. This enhancement is attributed to the protective layer provided by Al_2_O_3_, which maintains the constancy of LMO during charge–discharge reactions [88,89]. LMO (LiMn_2_O_4_) possesses a high theoretical specific capacity of approximately 148 mAh g^−1^ and an operating voltage of about 4.1 V vs. Li/Li^+^, making it a promising cathode material for lithium-ion batteries. LMO becomes unstable over time, mainly because of changes in Mn^3+^ ions and manganese dissolving. This causes the battery’s capacity to drop by about 30% after 200 cycles at room temperature. To overcome this limitation, surface modification strategies such as Al_2_O_3_ coating (typically 2–10 nm thick) have been widely investigated. Studies report that Al_2_O_3_-coated LMO electrodes can achieve capacity retention of about 95% after 200 cycles, compared with 80% for uncoated LMO. The coating layer also decreases charge-transfer resistance from ~200 Ω to 90 Ω, while suppressing Mn dissolution into the electrolyte. Lithium-ion movement across the interface and surface profile between the lithium metal and Li-Mg alloy electrodes in solid-state batteries is significant. These considerations determine the extent to which a battery performs well, its stability, and the extent to which it can be charged and discharged [90,91,92]. Lithium metal electrodes have disadvantages, including slow self-diffusion and void formation during stripping, which restrict their power density. In comparison, Li-Mg alloys have a higher level of morphological stability and in some cases, they can enhance lithium delivery and cycling properties. The self-diffusion of Li in pure Li metal is too low to maintain high current densities, resulting in the development of voids on the interface with solid electrolytes. The shape of the lithium electrodes after cycling may develop particulate and needle-like morphology, and the latter will result in the so-called dead-Li and loss of capacity. Li–Mg alloys exhibit improved surface morphologies owing to the processing conditions, which influence their electrochemical properties [93].

**Figure 4:**
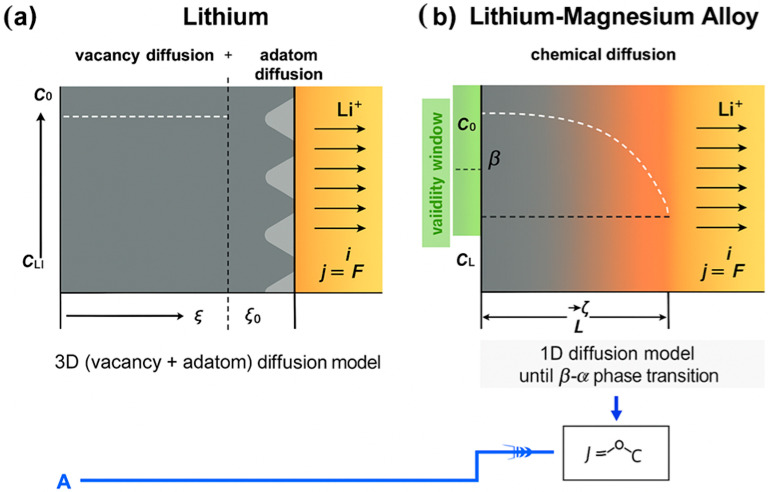
Schematic summarizing the lithium transport properties and morphological change at the interface for (**a**) a lithium metal electrode and (**b**) a Li-rich Li–Mg alloy electrode during anodic load [94].

Figure 4 shows how lithium moves and how the surface changes when stress is applied. It compares (a) a pure lithium metal electrode and (b) a Li-rich Li–Mg alloy electrode. In both cases, ξ is the distance from the back of the electrode, CLi is the amount of Li in a specific area, and L is the total thickness of the alloy. For pure lithium metal (a), the diffusion of vacancies and adatoms resulted in a narrow structural relaxation zone with a thickness of ξ_S_ near the active interface. In contrast, the Cd-Li Mg alloy electrode (b) exhibited chemical (bulk) diffusion, which balanced the concentration gradients across the electrode. Blue line indicates transition to simplified one-dimensional diffusion model after phase change; A marks starting reference point of analysis. The Li concentration gradually decreased to C0–CLi, and no structural relaxation region was observed, indicating the formation of fewer pores. This uniform diffusion in the alloy minimizes changes in the interfacial morphology, enhances surface stability, and reduces dendritic growth compared to pure lithium metal [94].

The interface between the lithium metal and solid electrolytes can be improved by developing interlayers that facilitate lithium transport and inhibit dendrite growth, as shown by the Zn(NO_3_)_2_-induced interfaces. The Al_2_O_3_ coating was applied using atomic layer deposition, where Al atoms from the precursor occupied interstitial sites on the LMO surface, lowering the oxidation state of Mn ions and boosting the electrochemical capacity [95]. Although the Al_2_O_3_ coating greatly enhances the LMO performance, further improvements can be achieved by adding elements such as Ni, which has been found to stabilize the structure and further improve the capacity retention. Ni-r30% cathodes with tungsten oxide and ZrO_2_ coatings are very effective in enhancing long-term cycles, and surface degradation is minimized; however, hybrid and conformal coatings of all-solid-state batteries solve interfacial instability and enable high-voltage operation. This field is rapidly evolving, with ongoing research focused on optimizing coating composition, thickness, and deposition techniques [96].

Studies have established that coating surfaces is a highly efficient method of improving the output of battery electrodes. There is considerable evidence of improvements in the electrochemical stability, cycling life, and capacity retention of different battery types. Nickel-based cathodes have shown the greatest gains, and in this case, coatings such as ZrO_2_, WO_3_, and hybrid surfaces have proven beneficial in mitigating degradation and capacity loss, even at high voltages and temperatures [97]. Coatings in all-solid-state and sodium-ion batteries are important at the interfaces of all-solid-state batteries and for enabling practical operations. However, there remains a problem in maximizing the thickness, uniformity, and compatibility of coatings with changing battery chemistries. Regardless of these complications, this field is advancing rapidly, and hybrid and multipurpose coatings are promising developments in battery technologies [98].

Surface coating of electrode materials is an established and versatile technique for enhancing the electrochemical performance, cycling, and capacity retention of batteries. This method is particularly well suited to Ni-rich cathodes in lithium-ion batteries, although it can also be used in other chemistries, including sodium-ion and all-solid-state batteries. Recent studies have focused on perfecting coating materials, methods, and scalability to meet the demands of future energy storage solutions [99]. Furthermore, more systematic research is needed to compare coating methods for various types of electrode materials and battery types.

### 3.3. Solid Oxide Cells

The development of thermal spray multilayer ceramic structures for applications in solid oxide cells (SOCs) is a potential area of research aimed at improving the performance and lifetime of such cells. Such structures are made up of various layers of ceramics, each with a specific purpose, e.g., ionic conduction or mechanical stability. The motive behind this strategy is to enhance ionic and diffusivity, minimize thermal expansion disparity, and enhance the performance of the whole electrochemical cell. Zirconia–yttria (YSZ) is one of the most frequently 95% used electrolyte materials owing to its 80% high ionic conductivity. It is commonly combined with lanthanum strontium manganite (LSM) to yield co-sintered structures that exhibit the best density versus porosity [100].

**Figure 5:**
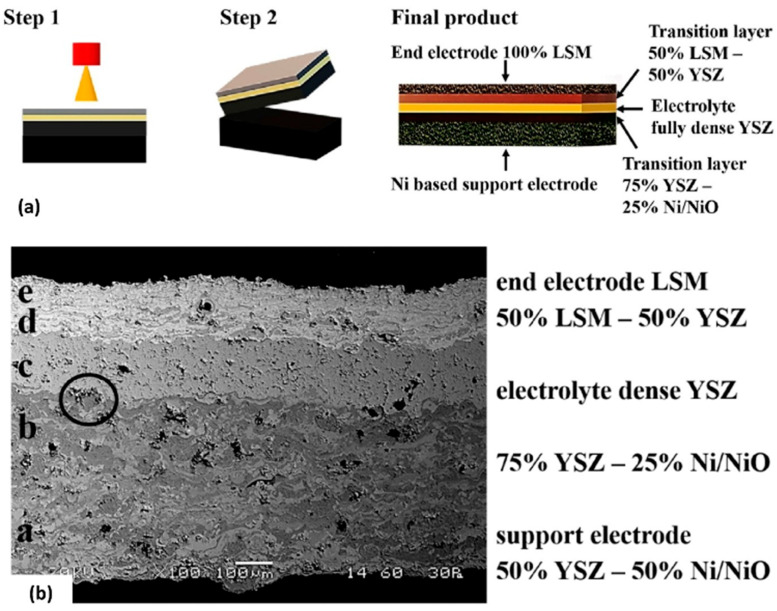
Multilayer ceramic coatings: (**a**) Multilayered structures formed during thermal spraying and, (**b**) cross-section of the coating [101].

Figure 5 (top) shows the two-step thermal spray procedure used to manufacture a free-standing solid oxide cell. In the second step, a multilayer stack was deposited onto the substrate, resulting in a self-supporting cell. The region represented in a circle is a porosity or voids formed during processing. The cross-sectional micrograph shown below illustrates the bonding of the layers (labeled a–e based on support, transition, electrolyte, transition, and electrode, respectively), which are approximately 50 µm thick. Graded compositions played a significant role in reducing thermal and mechanical discrepancies, and a dense YSZ electrolyte provided gas impermeability. This architecture takes advantage of the potential of the thermal spray method for depositing thick, functionally graded surfaces with one scalable process [101].

Despite the growth in the development of multilayer ceramic-based structures of solid oxide cells (SOCs), which have shown significant potential, various issues still exist, especially in establishing an optimal balance between mechanical stability and electrochemical functionality. The combination of metal supports and thin-film electrolytes has been a source of research attention with the aim of improving the start-up and thermal cycling speed of SOCs [102]. Tailoring thermal spray parameters for these materials is crucial to achieving optimal coating microstructures and performance in battery systems.

## 4. Process–Structure–Performance Relationships

Thermal spray coating is an advanced surface engineering technique that directly influences the process–structure–performance relationship in electrochemical energy devices. This is achieved by spraying feedstock material in a molten or semi-molten state onto substrates to create thick coatings with modified microstructures. The synergistic conversion of energy is achieved with photogene-generated carriers and thermal gradients supported by energy fields. External physical fields enhance battery performance. It examines the regulations governing these areas in the electrochemical processes. The research also provides concepts and methods for creating high-energy storage systems and enhancing renewable energy technologies [103]. The manner of putting the coating together is quite important in making it more effective, particularly in the way the layers are adhered. The bonding rate of a typical thermal spray coating is approximately 32%; therefore, it is not very strong or durable. However, by making changes in the spray parameters, such as the temperature of the particles and heating of the surface to be coated, the bonding rate can be improved to 100%. This will result in enhanced mechanical properties, and the grains can grow in a manner that makes the coating denser and more stable [104].

The microstructure and mechanical properties of the coating are highly sensitive to the processing conditions, including the feeding stock composition of thermal sprays, the state of the spray particles, and post-processing treatment. The incorporation of laser treatment before, during, or after thermal spraying can be used to modify the coating microstructure, enhance the adhesion to substrates, alter the phase composition, and improve wear and corrosion resistance without compromising environmental safety. Laser pretreatment enhances the adhesion between the coating and substrate by topographical texturing and ablating contaminants; laser-enhanced in situ melting during spraying alters the crystallinity and density of the coating layers; and post-laser treatments result in the creation of phases and structural refinement [105].

Thermal spray coating can be significantly enhanced in terms of structure, hardness, and wear and corrosion resistance by post-processing techniques, such as friction stir processing. This plays a role in preserving electrodes when in hard chemical environments. The re-crystallized microstructure and the increased mechanical strength reduce the degree of degradation mechanisms thereby increasing the lifespan and the performance of the batteries. Sophisticated sensing and control techniques in the thermal spray processes have improved reproducibility and the in situ measurement of coating characteristics such as, modulus and residual stress. These new techniques improve the structure and strength of the coating in real time. This is required to get energy devices to perform optimally [106,107].

Table 3 reveals that, although thermal spray research typically discusses the characteristics of coatings, such as porosity, hardness, adhesion strength, and phase composition, they should be interpreted differently when applied to batteries. They should be viewed in light of their impact on the performance of batteries, and include cycling stability, rate capability, interfacial resistance, ionic transport, and long-term electrochemical stability. To scale the coating design to any practical battery operation, it is crucial to identify a straight correlation between the coating microstructure and electrochemical response.

## 5. Cross-Cutting Challenges

By using thermal spray coating of the electrodes when preparing batteries, the porosity, phase, and crystalline formations can be tailor-made, with potential enhancement of the electrochemical results. It is very hard to get the right microstructure in these applications. This is because thermal spray processes are very sensitive to many factors. These include the type of plasma or gas used, the temperature and speed of particles, the distance and angle of the spray, and the properties of the feedstock. These factors can cause defects in the microstructure, which can affect how well the device works and how long it lasts [113].

### 5.1. Porosity and Gas Tightness

Plasma-sprayed coatings tend to have lamellar porous structures with unbound interfaces and microcracks, which may cause gas leakage and low ionic conductivity. The formation of a strong, fault-free microstructure is not an easy task because of the rapid formation of solidifications and splats during spraying. The most favorable porosity contains diffusion channels for gas and has adequate triple-phase boundary (TPB) areas of reaction. With low porosity, the TPB density rapidly increases with porosity up to a percolation threshold of about 10%. North of this, a balance of the surface area between the phases rather than the volume fraction is critical to establishing an optimal TPB density. An increase in the porosity (>25%) may cause a decrease in the TPB density due to shrinkage and detachment of the Ni/YSZ interface [114].

**Figure 6:**
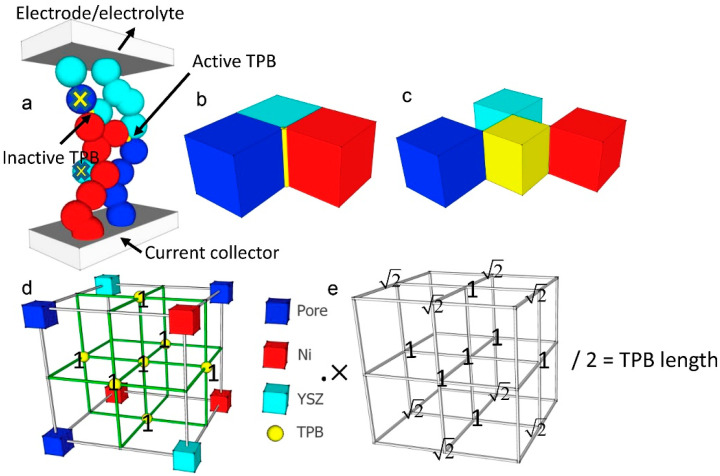
Schematic of triple phase boundary formation and modeling: (**a**) active/inactive TPB, (**b**,**c**) phase interfaces, (**d**) 3D microstructure network, and (**e**) geometrical estimation of TPB length within unit cell [115].

Figure 6 shows a method to measure active triple-phase boundaries (TPBs) in materials. This is important for electrochemical devices. Part (a) shows a 3D network of particles. It separates active TPBs from inactive ones. The inactive TPBs are marked for removal. The three constituent phases (b and c) are shown expanding by incorporating interstitial lattice points, which result in potential sites (in yellow) where the TPBs can be located, where all three phases meet. Part (d) is an example of how the TPBs are arranged spatially in terms of the location of the other phases in their environment with critical points of intersection. In part (e), there is a calculation matrix which assigns the distance in Euclidean distance in relation to a central node, and using this matrix to all TPB points and summing the products results in the total TPB length. Colors denote phases: red (Ni), cyan (YSZ), blue (pore), yellow (TPB). Cubes show phases, spheres particles. Numbers (1, √2) represent edge and diagonal lengths for TPB calculation. This method enables the active TPB length to be accurately and automatically measured, which is critical in improving the electrochemical performance [115].

Over-densification of Ni decreases the TPB density, resulting in a decrease in the TPB length and an increase in the polarization resistance. The quality of lanthanum strontium titanate (LST) and lanthanum strontium manganite (LSM) tubes largely relies on the quality of their coating and gas tightness in applications such as mechanical seals. Laser surface texturing is used to increase the gas-sealing of LST tubes by maximizing the surface characteristics to increase their sealing characteristics [116,117].

**Figure 7:**
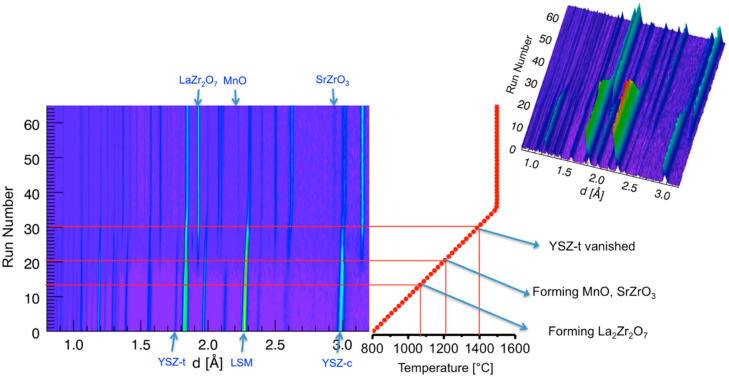
Neutron diffraction contour plot of the 3YSZ-LSM reaction and phase transformation during heating between 800 and 1500 °C for 5 h [118].

Figure 7 presents a neutron diffraction contour plot showing the phase evolution of a 3YSZ–LSM system during heating from 800 to 1500 °C with a 5 h holding period. The horizontal axis represents the diffraction parameter d (Å), and the vertical axis indicates the run number, corresponding to a progressive temperature increase. Initially, strong diffraction peaks of YSZ-t (tetragonal yttria-stabilized zirconia) and LSM (La–Sr–MnO_3_) are observed. The YSZ-t peak slowly decreases with increasing temperature, implying a structural transformation and interaction with LSM. The red dotted line with circles represents the temperature-dependent evolution path across sequential runs. Interfacial reactions and phase formation are confirmed by the emergence of new peaks that are related to La_2_Zr_2_O_7_, MnO, and SrZrO_3_. The inset (3D) shows the variation in the intensity with temperature, which indicates a gradual change in the phase with higher-temperature exposure [118].

LSM tubes are designed to maintain the tightness of the gas and withstand high temperatures. The use of LSM as a coating substance is crucial in reducing the oxidation rates, which are crucial in maintaining the tightness of gases over time.

### 5.2. Interfacial Bonding and Mechanical Integrity

Microstructural defects at the interface of thermal spray coatings are known to compromise the efficiency of electrochemical energy systems and, to a large extent, most of the time. One issue is poor splats bonding. This means the splats do not stick together well. They lack strong connections, which makes it hard for electrons to move. This lowers electrical conductivity. Poor bonding generally occurs when the particle temperature during deposition falls below the optimal melting range of approximately 2200 °C for refractory ceramics or when oxidation develops between successive layers with oxide thicknesses of 200 nm. This results in smaller contact areas, usually less than one-third of the total interface, during the deposition of refractory ceramics [119]. These issues are aggravated by the existence of microcracks, which offer more channels of electrical resistance. These flaws are both intra- and inter-splat cracks, which arise due to the quick cooling and thermal expansion dissimilarities and interrupt the flow of conductive lines. Changes in the tiny cracks and holes in a material can change how it conducts electricity. This can lead to large differences in how we measure its resistance. The interrelation between these defects creates a network of resistive paths with high resistance thus enhancing the ohmic resistance of the structure of coating layers considerably [120].

**Figure 8:**
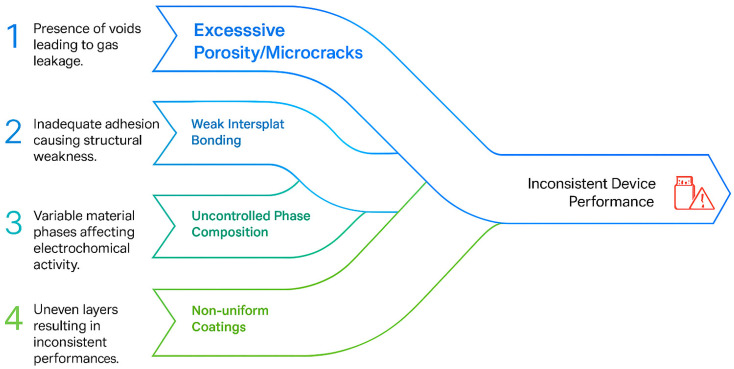
Microstructural challenges and device performance.

Figure 8 shows that materials with high porosity, weak bonds between layers, and uncontrolled phase composition and coatings lead to devices performing inconsistently. This is due to structural weaknesses and different electrochemical activities.

The mechanical strength decreases because weak inter-splat bonding undermines the ability of the coating to handle mechanical and thermal loads. Research shows that weak bonding between layers causes them to come apart when under stress. This is especially true in high-stress situations, where the wear can be three and a half times more than in well-bonded layers. The lamellar nature of thermal spray coatings, combined with the low bonding between splats, results in a favored failure mode: spreading under cyclic loads. Machinery frailty diminishes the existence of instruments. This is because the coating is not very useful in preventing corrosion, wear and changes in temperature. Consequently, the material at the bottom degrades prematurely, which influences the overall performance of the device [121].

Decreasing the temperature during thermal spraying at extremely high or extremely low temperatures may result in incomplete crystallization, phase segregation, or uneven phases. This influences the efficacy of the material in the electrochemical processes. The preciseness of the spray parameters (particle temperature and velocity) is important to achieve the desired phase and microstructure. The microstructures of large or complex surfaces are not uniform in advanced battery structures or three-dimensional microstructures. The thickness of the coating and its composition can be different, so that the performance could be uneven [122]. To chemically optimize the microstructure of the thermal spray coatings in batteries, it is important to regulate the deposition and post-treatment parameters and to select the material heterogeneously. Coprimary of these challenges is the need to improve field efficiency, periods of use, and scalability of new-generation energy instruments.

## 6. Future Directions and Emerging Trends

More attention to the interface design will be given to thermal spray coating technologies of energy systems in the future. This is since low porosity and high contact area are desirable in minimizing resistance and battery life. Complex techniques will attempt to minimize detrimental holes at critical connectors. They will also maintain sufficient routes of navigation. This can be used to facilitate the movement of electrons and maintains the transfer of charge with time [123]. This control is necessary to minimize energy waste, check for local overheating, and avoid damage, such as layer peeling and crack development. From the desire of energy storage devices to have increased power and extended durability, the need to have evenly distributed currents and surfaces that are well connected will increase [124].

In Table 4, the collected values are a mixture of studies that are specific to the battery and the general literature on thermal spray. While certain parameters are directly reported in battery-related applications (porosity, adhesion strength, etc.), others are based on well-established thermal spray research on analogous material systems, as standardized battery-specific data are limited. Therefore, the reported ranges are normal values at optimized processing conditions and are designed to provide comparative values and not absolute values.

Functionally graded materials and high-entropy alloys are expected to play a central role in this evolution. FGMs have special changes in their makeup and strength. These changes help reduce problems from heat and stress. Thus, they are useful for new batteries that face different heat and chemical conditions [133]. HEAs stick well, are tough to break, and resist rust. This makes them good for keeping surfaces stable in harsh conditions. SPS and liquid precursor HVOF are new methods in processing. They allow precise control of structure and porosity without affecting bonding or strength. These methods support the development of coatings that simultaneously enhance ionic transport and structural durability [134,135].

More applications of cold spray technology in metal current collectors and conductive layers are likely to be used. This is because it produces coatings that are thick, free of oxides, or adhere well. The introduction of new resources and controlled process management, data observation, and data improvements driven by data will likely accelerate. These trends reflect a trend towards interface-oriented designs. Porosity, bonding, and structure should be controlled in order to develop effective and powerful energy systems [136]. Topics discussed in this review are categorized based on their level of experimental validation in battery systems. While nanostructured feedstocks are well-supported, concepts such as HEAs and energy-aware powder synthesis remain largely exploratory.

### 6.1. Advanced Coating Materials

In industry, the production of sophisticated coating materials has led to alterations in different applications, such as the aerospace and consumer electronics industries. These state-of-the-art coatings not only offer better corrosion, wear, and environmental protection but also offer a much longer life to products. Furthermore, with sophisticated coatings, unprecedented properties can be imparted; these include but are not limited to self-cleaning, antimicrobial, or heat-resistant properties, leading to new product properties and their subsequent design. Nanostructured coatings have emerged as a prospective technique for enhancing the surface properties in a variety of applications. The unique properties of nanostructured coatings can be attributed to their high surface-to-volume ratio and the ability to control the properties of the material at the nanoscale [137].

**Figure 9:**
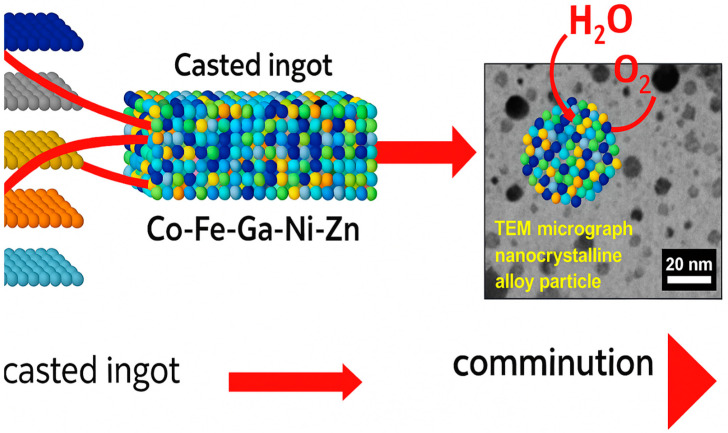
Schematic preparation of nanocrystalline HEA powder for the oxygen evolution reaction (OER) [138].

Figure 9 illustrates the systematic steps for producing nanocrystalline high-entropy alloy (HEA) powder to achieve the goal of oxygen evolution reaction (OER). This is achieved by the prudent selection of a set of transition metals (typically Fe, Co, Ni, Cr, and Mn) that have synergistic catalysis, as they have different electronic structures and binding affinities. Crystalline structures lead to face-centered cubic (FCC) or body-centered cubic (BCC) structures that form single-phase solid solutions and maximize atomic mixing entropy while maintaining structural stability. Surface optimization is a technique that involves controlled oxidation or reconstruction to generate active hydroxide/oxyhydroxide species (M-OOH), which are the primary catalyst sites for OER. The powder was characterized to demonstrate that the desired particle size distribution, compositional uniformity, and electrochemical performance were achieved to ensure oxygen evolution catalysis [138].

Functionally graded materials (FGMs) have recently become the topic of a lot of interest due to their peculiarities and possible applications. The smooth transition in the composition, structure or properties of FGMs across their volume allows them to perform on a custom basis in specific regions. This gradation can be accomplished using various manufacturing methods, such as powder metallurgy, centrifugal casting, and additive manufacturing [131]. A promising use of FGMs is the creation of self-healing coatings for the aerospace, automotive, and construction industries. These coatings may also have a layer that can heal when there is damage in the environment, like temperature [139]. The smart use of these aspects within the thickness of coatings allows a designer to develop a scheme in which the slightest cracks and scratches can be healed.

### 6.2. New Application Areas

New battery technologies are being created to fix the problems of regular lithium-ion batteries. These regular batteries usually provide an energy density of 250 Wh kg^−1^ and last for approximately 2000 cycles. New types like lithium–sulfur (Li–S), lithium–air (Li–O_2_), sodium-ion, and multivalent-ion batteries are promising for future energy storage. Li–S batteries can theoretically hold 1675 mAh g^−1^ and have an energy density close to 2600 Wh kg^−1^, which is much higher than current Li-ion batteries. However, they face issues such as the polysulfide shuttle effect, slow redox reactions, and a volume expansion of approximately 80%, which make them difficult to use practically [140].

Recent studies show that single-atom catalysts (SACs) are a big step forward in battery technology. They utilize almost 100% of each atom and have distinct active sites. This makes reactions faster and reduces unwanted reactions in metal–sulfur and metal–air batteries. SAC-based electrodes have improved catalytic activity by 2–5 times and have better cycling stability, especially in Li–S and Na–S battery systems [141].

Advanced batteries are being added to electric cars, power grids, and renewable energy systems. This will help them spread widely. Electric car batteries usually need to hold 120 kWh of energy. They should last for more than 3000 users and work well for 15 years. New materials and designs have led to flexible and wearable devices that can collect energy. These devices have power densities of 10 mW cm^−2^ and are good for portable electronics, medical devices, and smart clothing. Future energy systems will require storage technologies that are highly efficient (over 90%), last a long time, and use sustainable materials to meet global energy needs [142,143]. The future systems of energy demand state-of-the-art storage and conversion technologies to be efficient, reliable, and sustainable.

### 6.3. Artificial Intelligence for Electrochemical Energy Storage

Recent advances in artificial intelligence (AI) have played a major role in the design of materials for electrochemical energy storage systems (EESSs). The machine learning (ML) algorithms, data-driven methods, and feature engineering have allowed AI-based approaches to generate high-performance materials in high-rate fashion. The technologies in question contribute to the identification of new chemicals, the enhancement of the properties of materials, and accelerate the process of determining new materials. This can make the energy storage systems more efficient and effective. Applications of AI in material design include recoding existing systems and developing new technologies for energy storage [144]. The discovery of a multidimensional material space has been achieved with the help of AI and ML tools, as traditional methods are challenging to explore effectively. These tools not only result in high throughput discovery but also accelerate most of the operations of virtual screening and inverse molecular design. With feature engineering, alternative sets of material descriptors can be applied in the optimization of the material design and the enhancement of the performance of EESSs [145]. Simulations that involve multiple physical processes (so-called multi-physics simulations) are challenging and offer opportunities in science and engineering. Such simulations are essential to the adequate modeling of complex systems in the real world, but they need advanced computational processes and infrastructure. Some of the major challenges are linked with the necessity to coordinate different physical models, computational complexity and optimization of high-performance computing (HPC) systems. There are future chances to solve these problems and improve multi-physics simulation skills by advancing software and computational methods [146]. When these conditions were found at the junction of various scales of calculations, they were subjected to another level of computation. This assisted in knowing the form of the hydride inclusions [147].

## 7. Computational Modeling and AI-Driven Design of Thermal Spray Coatings

The integration of computational modeling and artificial intelligence (AI) has emerged as a powerful approach for accelerating the design and optimization of materials used in electrochemical energy systems. In the context of thermal spray coatings for batteries and solid oxide cells, these tools provide atomistic to system-level insights into material behavior, interfacial stability, and performance prediction.

### 7.1. Density Functional Theory (DFT) and Atomistic Modeling

Recent developments in ab initio and density functional theory (DFT) have aided the understanding of materials at the atomic scale in electrochemical environments. This involves the motion of ions, interface stability, and electronic structure. Such approaches are increasingly applied to predict major performance parameters of electrochemical energy storage systems, such as voltage profiles, diffusion barriers, and phase stability [148]. Moreover, DFT modeling of doped oxide systems is important for critical studies on defect chemistry, electronic conductivity, and surface reactivity, which are critical in the design of coatings for battery electrodes. New techniques based on basic estimations and machine learning have recently been developed. These techniques are useful in discovering and enhancing solid-state electrolytes and coating materials far more rapidly [149,150].

Thermal spray is becoming a good way to create coatings for new batteries. However, the design tools used are mostly from general thermal spray and battery communities, not specifically for battery coatings. Hybrid SPH-finite element models are useful in investigating impacts of droplets impacting surfaces, splat splats, temperature variations, and residual stress in ceramic coating. These models can forecast the microstructure and stress that may be applicable to battery layers [151,152].

### 7.2. Multiscale Modeling and Process–Structure–Performance Correlation

Multiscale modeling methods, other than atomistic simulations, are useful for combining the physics of thermal spray processes with the development of coating microstructure and electrochemical battery performance. The modeling of the behavior of particles in thermal spraying relies on simulations of particle temperature, velocity, and in-flight behavior, which is based on computational fluid dynamics (CFDs) and particle dynamics. The models for the Diamond Jet hybrid HVOF process show that particle speeds are usually around 900 m s^−1^. The particle temperatures can reach 3000 K. These rely on the ratios of fuel to oxygen and the distance of the source. These parameters have a strong impact on the melting conditions of particles before collision and the morphology of splats [153].

On the scale of interaction of particles with surfaces, a combination of computer approaches is employed. Such techniques are smooth particle hydrodynamics (SPHs) of droplet impact and finite element (FE) of heat transfer and shape change. Collectively, they assist in the interpretation of the formation, spreading, rapid drying, and acquisition of stress in thermal spray coatings. Recent experimental and multiscale studies are very strong in supporting these models. Research on the effect of heat and force on APS coatings showed that their computer simulations were equal to the real-life stress measures [154]. These stresses influence the ability of the coating to stick and the development of cracks. These stresses arise from the rapid solidification process and thermal mismatches and are closely related to the splat–scale deformation modeled using SPH–FE [155].

The results of these tests indicate that small-scale processes, such as spreading, creating tiny holes, and residual stress, are the major contributors to the structure and strength of the coating. These characteristics influence the quality of the coating by altering the electrically flowing, ionic movement, and resistance at the surface. This establishes a direct connection between process, structure and performance of the energy systems [156].

### 7.3. Artificial Intelligence and Machine Learning Approaches

The use of AI and machine learning is becoming increasingly common to accelerate the discovery of new materials and the enhancement of the processes of thermal spray coating. According to recent research, they are effective. These data-driven models are useful in the rapid verification of coating materials and their tendency to influence how the structure influences their properties. As an example, experimental data, when applied to the models of random forest and neural networks, can predict the coating porosity and hardness of the atmospheric plasma spray systems. These have more than 90% accuracy and are also comparable to real measurements [157]. Electrochemical performance could be associated with process settings using machine learning techniques, such as neural networks and gradient boosting models. It is found that the use of ML to enhance thermal spray coatings may be used to increase electrical conductivity and reduce interfacial resistance [158].

This has a direct result of improving battery performance and stability. This study investigates the fabrication of reversible metal-supported solid oxide cells (RSOCs) and their performance. These cells are created using atmospheric plasma spraying (APS) at the National Atomic Research Institute (NARI). The RSOC design included a three-layer composite electrolyte composed of Sm-doped ceria (SDC) and Sr− and Mg-doped LaGaO_3_ (LSGM). This design improves ionic conductivity and lowers polarization resistance. The 10 × 10 cm^2^ cells produce peak power densities of 660, 798, and 859 mW cm^−2^ at temperatures of 650, 700, and 750 °C, respectively, when operating at 0.7 V in fuel cell mode. These findings indicate that RSOCs fabricated using APS are an excellent choice for efficient and long-term conversion and storage of energy at medium temperatures [158].

AI control systems in real time make it possible to make changes to the spray conditions on the spot. This enhances the even coating and minimizes flaws in the application process. New materials such as high-entropy alloys (HEAs) and functionally graded materials (FGMs) are also designed with the use of AI. The optimal multi-component compositions are located by means of rapid computational screening and machine learning. They are more phase-stable and corrosion-resistant, reducing trial and error experimentation. These works indicate that AI and ML are powerful tools. They assist in linking processing, microstructure and performance [159]. This aids in the development of long-life cycle high-performance coatings for new electrochemical energy systems.

### 7.4. Machine Learning Approaches

A promising approach is to combine DFT with machine learning. Here, data from basic calculations is used to train models that can make predictions. Integrating machine learning with conventional computational techniques can significantly improve the simulation speed and predictive performance. Studies have shown that machine-learning-assisted simulations can reduce computational time by approximately 90% while maintaining prediction accuracies of 98% compared with traditional physics-based models. It will need ongoing research and development as the field advances to address these challenges and make the most out of multi-physics simulations [160]. This process produces a fully digitalized structure annotated with component identities or a particle dataset ready for statistical analysis, facilitating the rapid quantitative [161]. Currently, there is no comprehensive physics-based model that allows for the understanding of the thermal behavior of capacitors. This type of model should consider the thermophysical properties of all materials that are completely used and the heat generated by all sources. Therefore, the creation of new effective cooling solutions to avoid overheating and mitigate associated complications, including the aging effect and performance degradation, can be achieved [162].

Further testing is required to better understand the aging and depreciation of electrolyzers under different operating conditions. High temperatures in electrolyzers usually lead to an increase in the electrochemical efficiency, but may also accelerate the rate of degradation. Before trying to identify the optimum operating conditions for a range of different scenarios, it is useful to know the rate of aging and degradation [163].

Even with these advances, combining ab initio predictions with thermal spray process settings and changes in coating structure is still a new research area. This is especially true for predicting how process, structure, and performance relate in battery coatings.

## 8. Conclusions

This review defines thermal spray coating as an electrochemical energy systems process–structure–property–performance driven de-design approach, but not an erosive shield. By adding control over the microstructure of coatings, battery-relevant measures of performance, and computational design approaches and strategies, thermal spray technologies have become the enabling technology for a robust, high-performance, and scalable energy storage ecosystem.

1. Thermal spray is used to enhance the performance of batteries. They assist in the stability of cycling, rate ability, interfacial resistance, ionic transport and long-term electrochemical stability. This is achieved through the enhancement of the electrode–electrolyte interfaces even when subjected to harsh conditions.
2. When porosity is around 10–30%, it helps ions move better. Strong bonds between splats, over 70 MPa, make the material last longer and work well over time.
3. More recent materials, including high-entropy alloys (HEAs), functionally graded materials (FGMs), and nanostructured feedstocks, exhibit improved stability, stress resistance, and enhanced electrochemical reactions. These properties are useful in the creation of new interfaces and electrodes.
4. Microstructural details affect outcomes like lower resistance, better charge transfer, and improved capacity retention.
5. It is significant to use energy prudently when making coatings. It is the process of the trade-off between energy expended in powder production and implementation. This has an influence on the quality of the coating, the way its structure varies, and the effectiveness of the functioning and durability of the device.
6. New computer and data methods, like ab initio modeling, density functional theory (DFT), and machine learning, help predict how materials will behave.
7. Artificial intelligence, enabled by multiphasic simulations, enhances thermal spray coating. It enables real-time modifications, minimizes errors, and simplifies scalability. This method reduces trial-and-error experiments and ensures uniformity of findings.
8. Remaining challenges include minute defects (such as excessive holes, cracks, and weak links), variations in the process, and restrictions on the quantity that can be produced. These problems may damage the stability of connections and the lifetime of electrochemical systems.
9. Future studies are warranted to investigate interface improvements, different scales of models, and combinations of different coatings. This can be achieved via thermal spraying with advanced thin-film methods for enhanced control over the structure and functionality of materials.
10. Lithium-ion, sodium-ion, solid-state batteries, and solid oxide cells are new energy systems that are enhanced by thermal spray coatings. They render these energy appliances more efficient, durable and environmentally friendly.

## Figures and Tables

**Table 1:** Role of thermal spray methods in electrochemical energy systems.

Technology /Method	Electrolysis Type	Key Findings	Splat Bonding/Interfacial Integrity Implications	Opportunities
Comprehensive Review—APS, VPS, HVOF, SPS	Alkaline, PEM, SOEC	Thermal spraying facilitates the large-scale manufacturing of electrolyzer components, thereby enabling precise control over their microstructure and porosity.	The quality of splat bonding depends a lot on the temperature and speed of the particles. VPS and SPS help the splats stick together better and reduce oxide layers, which improves the connection at the interfaces.	Scalable manufacturing, cost reduction, advanced materials development, and hybrid approaches [16,17,18].
SPS, HVOF, Cold Spray	Alkaline Water Electrolysis	Nickel electrodes featuring three-dimensional fin arrays demonstrated low overpotentials and high current densities in alkaline environments.	High-kinetic-energy techniques, such as HVOF and cold spray, produce intense bonds because the materials are tightly interlocked. At an extremely small design, SPS assists in attaching materials more effectively.	Enhanced surface area, improved catalytic activity, and cost-effective manufacturing [19,20,21].
Vacuum Plasma Spraying (VPS)	PEM Electrolysis	VPS Ti coatings applied to stainless steel demonstrated excellent conductivity and corrosion resistance for use in bipolar plates.	Controlled atmosphere prevents oxidation, leading to high splat substrate bonding as well as interfacial resistance.	Cost reduction through stainless steel substrates, improved corrosion resistance [22].
Thermal Spraying-Sintering	SOEC	The thermal spray-sintering technique produced gas-impermeable electrolyte layers with a power density of 0.73 W/cm^2^.	Inter-splat bonds are filled in by post-spray sintering, interfaces are densified, and interlayer bonding is much enhanced.	Single-step processing, reduced sintering requirements, material conservation [23,24].
Various Thermal Spray Techniques	Alkaline, PEM, SOEC	Thermal spraying offers benefits for mass production in energy storage applications owing to the versatility of materials.	The area linkage can be strong or not. If the process is not regulated, it may lead to weak bonds and small cracks.	Industrial scale-up, novel material combinations, process integration [25,26].
Atmospheric Plasma Spraying (APS)	Alkaline Water Electrolysis	NiAlMo coatings applied via plasma spraying exhibited performance comparable to that of noble catalysts in facilitating oxygen evolution.	APS may experience splat bonding and oxidation; therefore, optimizing the spray parameters is important to maintain electrical continuity.	Noble metal replacement, improved durability, automated production [27].
HVOF Spraying	PEM Electrolysis	HVOF titanium coatings with a porous structure exhibited porosity levels ranging from 24% to 40% for transport layers in PEM electrolyzers.	The splat bonding is excellent because of high particle velocity regardless of porosity and the mechanical integrity.	Manufacturing cost reduction, improved mass transport, and automated processing [28,29].
Atomic Layer Deposition (ALD)	Batteries, PEM	Ultrathin conformal coatings (e.g., Al_2_O_3_, TiO_2_) improve interfacial stability and suppress degradation.	Atomic-level uniformity ensures excellent interface control without splat formation; strong chemical bonding dominates.	Precise thickness control, improved cycling stability, interface engineering [30].
Chemical Vapor Deposition (CVD)	Batteries, SOFC	Produces dense, uniform coatings with controlled composition and crystallinity.	Strong chemical bonding to the substrate; minimal defects but possible thermal stress owing to high processing temperatures.	High-quality thin films, improved conductivity, scalable for thin coatings [31].
Polymer-Based Coatings	Batteries	Flexible coatings enhance electrode stability and accommodate volume changes.	No splat bonding; the connection is controlled by how well the adhesive and polymer work with the electrode. It might have weak mechanical strength.	Flexible devices, low-cost processing, improved safety and stability [32].

**Table 2:** Comparison of thermal spray coatings.

Method	Best Use	Advantage	Limitation	Battery Metric Impact
APS	Cathode coatings, protective oxide layers	High versatility, scalable, controlled porosity	Oxidation, microcracks	Improves ionic transport and rate capability; may increase interfacial resistance if oxidation occurs [53,54,55]
HVOF	Current collectors, transport layers	Dense, Strong bonding	Lower porosity limits ion transport	Enhances electrical conductivity and reduces interfacial resistance; improves power density [56,57]
Cold Spray	Metallic conductive layers, anode protection	No oxidation, high adhesion	Limited ceramic deposition	Improves cycling stability and mechanical durability; reduces delamination [58,59].
SPS	Nanostructured electrodes, high-surface-area cathodes	High surface area, nano-features	Complex parameter control	Enhances electrochemical activity, ionic transport and reaction kinetics [60,61].
LPPS (Low Pressure Plasma Spray)	Solid oxide cell electrodes, bipolar plates	Controlled atmosphere, dense coatings	High cost, complex setup	Reduces interfacial resistance; improves long-term stability and corrosion resistance [62,63].
SPPS (Solution Precursor Plasma Spray)	Electrolyte layers, thin functional coatings	Fine microstructure, compositional control	Process instability	Enhances ionic conductivity and interface uniformity [64,65].
HEA-based Thermal Spray	Electrode stabilization, corrosion-resistant coatings	High durability, phase stability	Complex composition control	Improves long-term cycling stability and degradation resistance [66,67].
FGM Coatings (APS-based)	Interface layers (electrode/electrolyte)	Stress gradient control	Fabrication complexity	Enhances interfacial stability and cycling life [68,69].
Nanostructured Feedstock Spraying	High-rate electrodes	Enhanced kinetics, large surface area	Grain growth instability	Improves rate capability and ionic transport [70,71].
Suspension/Hybrid Spray	Advanced electrode architectures	Tailored microstructure	Equipment complexity	Optimizes ionic transport, interfacial resistance and cycling stability [72,73].

**Table 3:** Correlation between coating properties and battery performance.

Coating Property	Microstructural Feature	Battery Metric Affected	Mechanism
Porosity (10–30%)	Interconnected pores	Ionic transport, rate capability	Enhances electrolyte penetration but excessive porosity reduces conductivity [108].
Adhesion strength (>70 MPa)	Strong splat bonding	Cycling stability	Prevents delamination during volume expansion [109].
Phase composition	Stable crystalline phases	Long-term stability	Reduces degradation and phase transformation [110].
Surface roughness	High interface area	Interfacial resistance	improves the electrode–electrolyte contact [111].
Electrical conductivity	Dense splat network	Rate capability	Enables efficient electron transport [112].

**Table 4:** Enhancing battery durability and efficiency through thermal spray coating technologies.

Thermal Spray Technology/Application	Key Challenges	Dominant Battery Metric Affected	Dominant Battery Metric Affected	Mechanistic Interpretation
Atmospheric Plasma Spraying (APS)—Cathode/Anode Coatings	Oxide formation, weak inter-splat bonding, microcracking	Ionic conductivity; Cycling stability	Ionic conductivity; Cycling stability	High porosity improves electrolyte penetration, but weak inter-splat bonding reduces mechanical integrity [125]
High-Velocity Oxygen Fuel (HVOF)—Current Collectors, Transport Layers	Porosity control vs. mass transport, residual stresses	Interfacial resistance; Electrical conductivity	Interfacial resistance; Electrical conductivity	Dense coatings improve electron pathways and reduce contact resistance [126]
Cold Spray—Anode Protection Layers	Limited ceramic deposition, high residual stress	Cycling stability; Mechanical durability	Cycling stability; Mechanical durability	Strong adhesion and ductility prevent delamination during volume changes [127]
Suspension Plasma Spraying (SPS)—Active Cathode Layers	Nanostructure retention, phase stability	Rate capability; Ionic transport	Rate capability; Ionic transport	Nano-features shorten diffusion paths and increase active surface area [128]
Liquid Precursor HVOF—Functional Interlayers	Precursor decomposition, coating uniformity	Interfacial resistance; Stability	Interfacial resistance; Stability	Uniform coatings improve interface continuity and charge transfer [129]
High-Entropy Alloy (HEA) Coatings—Electrode Stabilization	Phase control, compositional segregation	Long-term cycling stability	Long-term cycling stability	High configurational entropy improves phase stability and suppresses degradation [130]
Functionally Graded Materials (FGMs)—Interface Layers	Gradient design complexity, process control	Interfacial stability; Stress tolerance	Interfacial stability; Stress tolerance	Gradients reduce thermal and mechanical mismatch at interfaces [131]
Nanostructured Ceramic Coatings—Protective Barriers	Grain growth, thermal instability	Ionic transport; Reaction kinetics	Ionic transport; Reaction kinetics	Fine grains enhance diffusion and electrochemical reactivity [132]

## Data Availability

No new data were created or analyzed in this study. Data sharing is not applicable to this article.
